# Combined Effect of Alternating Current Interference and Cathodic Protection on Pitting Corrosion and Stress Corrosion Cracking Behavior of X70 Pipeline Steel in Near-Neutral pH Environment

**DOI:** 10.3390/ma11040465

**Published:** 2018-03-22

**Authors:** Liwei Wang, Lianjun Cheng, Junru Li, Zhifu Zhu, Shuowei Bai, Zhongyu Cui

**Affiliations:** 1 College of Electromechanical Engineering, Qingdao University, Qingdao 266071, China; lijunru_qdu@163.com (J.L.); baishuowei1@163.com (S.B.); 2 Institute of Materials Science and Engineering, Ocean University of China, Qingdao 266100, China; cuizhongyu@ouc.edu.cn

**Keywords:** pipeline steel, AC interference, cathodic protection, pitting corrosion, stress corrosion cracking

## Abstract

Influence of alternating current (AC) on pitting corrosion and stress corrosion cracking (SCC) behavior of X70 pipeline steel in the near-neutral pH environment under cathodic protection (CP) was investigated. Both corrosion and SCC are inhibited by −0.775 V_SCE_ CP without AC interference. With the superimposition of AC current (1–10 mA/cm^2^), the direct current (DC) potential shifts negatively under the CP of −0.775 V_SCE_ and the cathodic DC current decreases and shifts to the anodic direction. Under the CP potential of −0.95 V_SCE_ and −1.2 V_SCE_, the applied AC current promotes the cathodic reaction and leads to the positive shift of DC potential and increase of cathodic current. Local anodic dissolution occurs attributing to the generated anodic current transients in the positive half-cycle of the AC current, resulting in the initiation of corrosion pits (0.6–2 μm in diameter). AC enhances the SCC susceptibility of X70 steel under −0.775 V_SCE_ CP, attributing to the promotion of anodic dissolution and hydrogen evolution. Even an AC current as low as 1 mA/cm^2^ can enhance the SCC susceptibility.

## 1. Introduction

With the increasing development of high voltage power transmission, its sharing common grid with oil and gas pipelines has brought huge risk to pipeline integrity [1,2]. It has been acknowledged that AC interference can speed up the corrosion rate of most metals [3], but the actual mechanism of AC corrosion is not clear. Effect of AC on polarization behavior of metals has been modeled theoretically by Lalvani et al. [4,5]. They related the AC caused change of corrosion potential and corrosion current density to the ratio between the anodic and cathodic Tafel slopes (indicated as *r* = *β*_a_/*β*_c_). In their theory, corrosion kinetic parameters, such as Tafel slope and exchange current density are supposed to be invariable with the interference of AC. Goidanich et al. [6,7] suggested that AC has strong influence on corrosion kinetic parameters depending on the system studied. Our previous work [8] showed that AC interference changed the specific adsorption of chloride ions, which can influence the pitting susceptibility of mild steel [9]. 

Recently, AC corrosion of pipeline steels has been paid much attention due to the numerous pipeline failure cases caused by AC interference [10,11,12]. Cheng and co-workers found that an applied AC would accelerate corrosion of pipeline steels in both high pH [13] and near-neutral pH environments [14]. The critical AC current densities to initiate pitting in these two solutions were approximately 300 and 200 A/m^2^, respectively. Du and co-workers investigated the influence of AC on the stress corrosion cracking (SCC) behavior of pipeline steels in high pH [15] and near-neutral pH solutions [16]. They found that AC interference changed the high pH SCC mode from intergranular to transgranular [15] and the near-neutral pH SCC susceptibility was enhanced by AC current [16]. However, the abovementioned works do not consider the cathodic protection (CP), which is mandatory for pipeline steels in the field [17].

Previous works [2,18,19,20] showed that AC affected the performance of CP system and caused deviation of CP potential from designed values. Hosokawa et al. [18] and Kajiyama et al. [19] reported that pipelines would suffer serious AC corrosion even if they satisfied the CP criterion potential of −0.85 V_CSE_ (CSE corresponds to the copper sulfate electrode). Xu et al. [20] found that the presence of AC would decrease the corrosion protection effectiveness of CP and the pipelines can be completely protected only when the CP potential was negative enough. On the contrary, Ormellese et al. [21] revealed that overprotection (potential more negative than −1.1 V_CSE_) seemed to be more dangerous than lack protection when pipelines are under AC interference. Nielsen et al. [22] also suggested that excessive CP increased AC corrosion risk of pipelines and should be avoided. Accordingly, influence of AC interference on corrosion of pipeline steels under CP has not reached consensus. Moreover, SCC problem under the combined effect of CP and AC has not been considered. 

In this work, effect of AC current on the electrochemical, corrosion and SCC behavior of X70 steel in the near-neutral pH environment under cathodic protection is investigated. The shift of CP potential and current by AC interference is measured and analyzed. Localized corrosion behavior including pitting and SCC are discussed.

## 2. Material and Methods

### 2.1. Material and Solution

**Figure 1:**
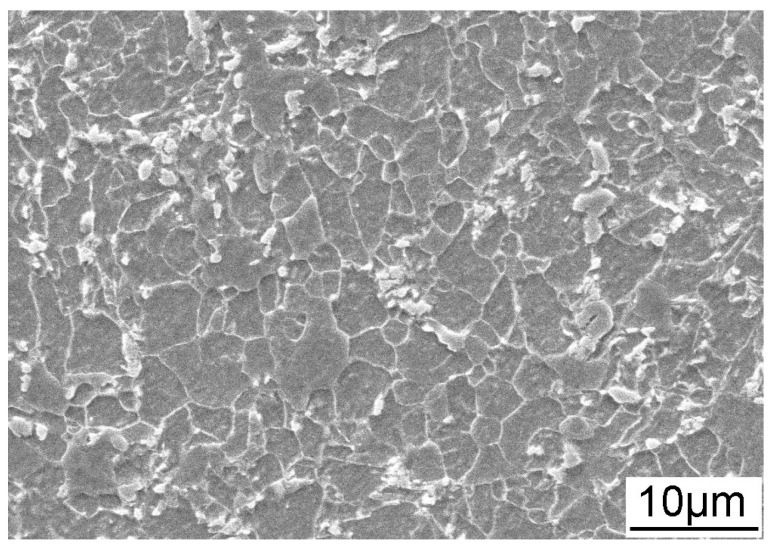
Scanning electron microscope (SEM) image of the microstructure of X70 pipeline steel.

The material used in this study was the as received X70 pipeline steel with the chemical composition (wt %) of: 0.065 C, 1.57 Mn, 0.23 Si, 0.2 Ni, 0.18 Cr, 0.22 Cu, 0.056 Nb, 0.002 S, 0.0019 P and Fe balance. Figure 1 shows the microstructure of the X70 steel, which mainly consists of massive polygonal ferrite and a small amount of pearlite, with fine M/A islands in the grain interior.

The test solution was the near-neutral pH (NS4) solution with the chemical composition of: KCl 0.122 g/L, CaCl_2_·2H_2_O 0.181 g/L, NaHCO_3_ 0.483 g/L and MgSO_4_·7H_2_O. This solution is the simulation solution of the electrolyte trapped under disbonded coatings. Before testing, the solution was purged with 95% N_2_ + 5% CO_2_ for 4 h to achieve an anaerobic and near-neutral pH condition (pH = 6.8). The gas flow was maintained throughout the test. All tests were performed at ambient temperature (about 22 °C).

### 2.2. Electrochemical Equivalent Circuit for the Experiment

**Figure 2:**
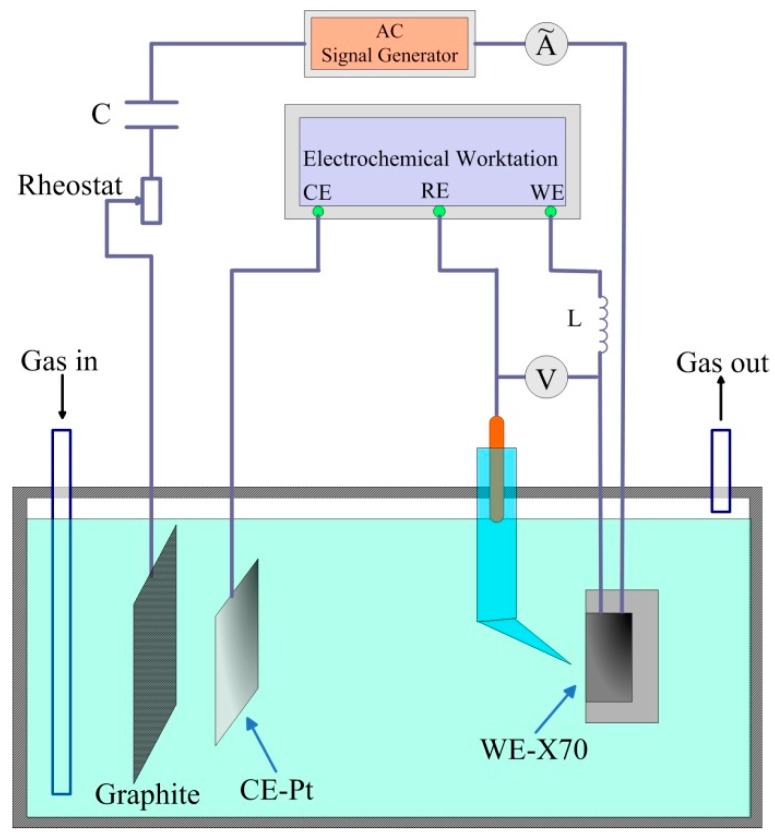
Schematic diagram of the experimental setup for the application of alternating current to the pipeline steel under cathodic protection.

Figure 2 shows the schematic diagram of the experimental set-up for studies of AC corrosion under cathodic protection. The electric circuit was specially designed to supply and measure AC and DC signals independently [6]. Ormellese et al. [21] and Büchler et al. [23,24] have used this circuit to investigate the alternating current corrosion of cathodically protected pipelines. In this work, an AT1645-3 function generator (ATTEN, Shenzhen, China) which was connected to the ground through its power supply was used to provide the AC signal. The sinusoidal AC signal with a frequency of 50 Hz was applied between the working electrode and a graphite electrode. A rheostat, with a range of 0–9999 Ω, was used to adjust the AC current to the present value. Within the AC mesh, an electrolytic capacitor (50 V, 470 μF) in series was used to prevent DC circulation. As described by Goidanich et al. [6], by using two electrolytic 1000 μF capacitors (total capacity of 500 μF and a capacitive reactance of about 6 Ω), the efficiency of the DC filter was 100%, i.e., no DC flowed in the AC mesh. Within the DC mesh, DC current was supplied by an electrochemical workstation and a 4H inductance with a series resistance of 76 Ω was introduced to avoid the interference of the AC signal to the electrochemical workstation. Despite the inductance, a small part of the AC flowed in the DC mesh, which is negligible for the tests planned. The root-mean-square value of the AC current was measured with a clamp meter. The experimental details were illustrated separately in the following sections.

### 2.3. Electrochemical Measurements

Specimens used for electrochemical measurements were coated with epoxy resin, leaving an exposure area of 1 cm^2^ as the working surface. The electrode was ground sequentially to 1500 grit emery paper, and then cleaned by acetone. The electrochemical tests were carried out through the Gamry Reference 3000 electrochemical workstation (Gamry, Warminster, PA, USA) using a three-electrode cell system, where the X70 steel was used as working electrode (WE), a saturated calomel electrode (SCE) as reference electrode (RE), and a platinum plate as counter electrode (CE, Figure 2). The distance between RE and WE was about 1 mm to reduce the ohmic drop in potential measurements. The steel was potentiostatically polarized by the electrochemical workstation to the cathodic direction and the variations of DC potential and DC current were measured. Simultaneously, the AC current was applied to the specimen through the AC circuit. In this work, the CP potentials of −0.775 V_SCE_, −0.95 V_SCE_ and −1.2 V_SCE_ were used to provide cathodic protection. The AC current densities of 1, 3, 5 and 10 mA/cm^2^ were imposed to the WE. During the tests, a Fluke 289C true RMS multimeter (Fluke Co., Everett, WA, USA) which had a high impendence was used to measure the potential between WE and RE. 

### 2.4. Immersion Tests

For the immersion tests, the samples were identical to those used in the electrochemical measurements, and the circuit in Figure 2 was used. Prior to the test, the specimens were wet ground with successive grades of silicon carbide abrasive papers from P120 to P2000 followed by diamond polishing to 0.1 mm. Then, the specimens were subjected to the DC/AC interferences for 2 h with DC potentials of open circuit potential (OCP), −0.775 V_SCE_, −0.95 V_SCE_, −1.2 V_SCE_ and AC current densities of 3 and 10 mA/cm^2^. After the test, the specimens were rinsed and dried carefully. Then, the corrosion morphology was observed via scanning electron microscope (SEM, Quanta 250, FEI, Hillsboro, OR, USA). For comparison, the immersion tests of the X70 steel under OCP and cathodic protection were also conducted, and the corrosion morphology was observed.

### 2.5. Slow Strain Rate Tensile Tests (SSRT)

**Figure 3:**
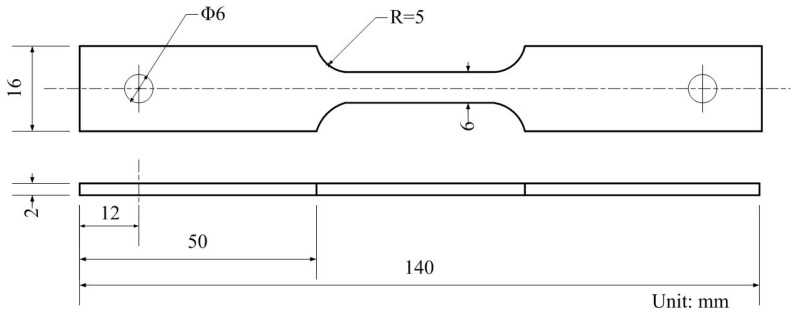
Geometry of the tensile specimen used in the slow strain rate tensile tests.

SSRT tests were used to characterize the SCC behavior of X70 steel in the near-neutral pH solution under CP and AC interference. The tensile test specimens were made according to GB T15970 specification [25] and the dimension of the specimens was shown in Figure 3. Prior to the test, the gauge length of the specimen was ground to 2000 grit emery paper along the tensile direction, then degreased with acetone and finally dried in air [26]. The specimen was soaked in the solution for 24 h before the SSRT test. During the process of SSRT test, the electrical circuit in Figure 2 was used to apply DC cathodic potential and AC current to the tensile samples. A basalt fiber was put in between the specimen and the pin to insure the insulation. Firstly, the SCC behavior of the cathodically protected X70 steel in the NS4 solution under the application of various CP potential without AC interference was investigated. The applied CP potential includes −0.775 V_SCE_, −0.85 V_SCE_, −0.95 V_SCE_, −1.05 V_SCE_, and −1.2 V_SCE_. For comparison, the SSRT tests of the X70 steel in air and under OCP without AC current were also conducted. Then, different AC current densities were superimposed to the −0.85 V_CSE_ (−0.775 V_SCE_) cathodically protected specimens. AC current densities of 1, 5 and 10 mA/cm^2^ were used. The SSRT strain rate of all tests was 1 × 10^−6^ s^−1^. All the tests were performed at ambient temperature (~22 °C) and repeated at least three times.

To investigate the SCC susceptibilities quantitatively, sensitivity index including loss of elongation (*R_δ_*) and reduction-in-area (*R_ψ_*) were calculated with the following equations:

(1) $$R_{\delta }=\left(1-\frac{\delta_{s}}{\delta_{0}}\right)\times 100\%$$

(2) $$R_{\Psi }=\left(1-\frac{\psi_{s}}{\psi_{0}}\right)\times 100\%$$

where *δ_s_* and *ψ_s_* are the elongation and the reduction-in-area in solution, respectively; and *δ*_0_ and *ψ*_0_ are the elongation and the reduction-in-area measured in air, respectively.

After SSRT tests, the fracture parts were cut off from the specimen and corrosion products on the fracture surface were cleaned in the HCl solution containing 1000 mL HCl, 1000 mL pure water and 3 g hexamethylene tetramine. After stirring in ultrasonic oscillator, cleaned by ethanol and then dried in air, the morphologies of the fracture surface were observed by SEM.

## 3. Results

### 3.1. DC Potential and DC Current Density Measurements

**Figure 4:**
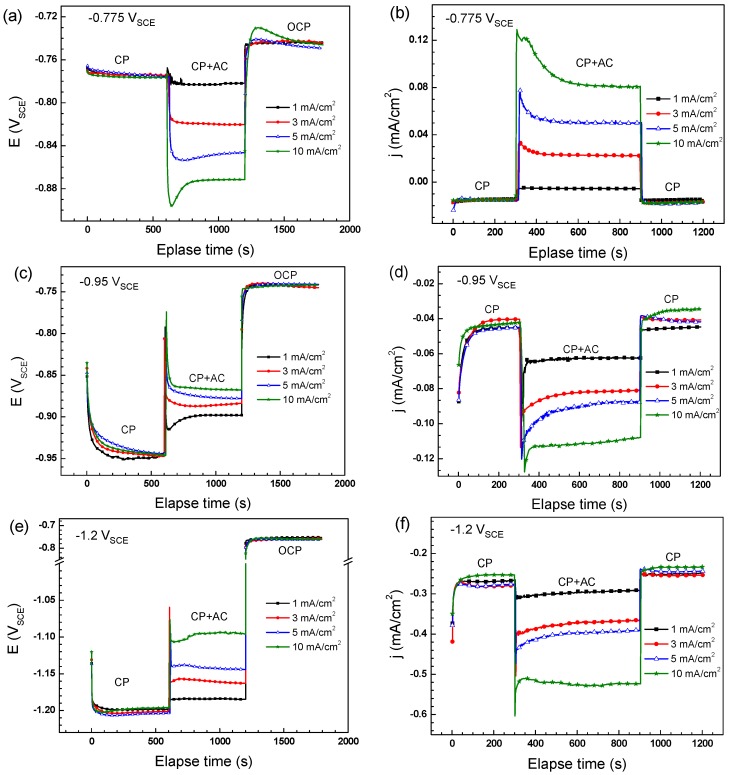
DC potential (**a**,**c**,**e**); and DC current (**b**,**d**,**f**) changes of X70 steel under the combined effect of AC interference and CP potential of: −0.775 V_SCE_ (**a**,**b**); −0.95 V_SCE_ (**c**,**d**); and −1.2 V_SCE_ (**e**,**f**).

The real-time DC potential and DC current density on the X70 steel at various AC current densities under different CP potentials were illustrated in Figure 4, and the corresponding steady-state values in the CP + AC region are listed in Table 1. For the DC potential measurements, each test is conducted in three stages and each stage lasts for 10 minutes. In the first stage from 0 to 600 s, the designed CP potentials are applied to the steel (CP region). Then, the AC interference is overlying to the CP protected steel in the second stage from 600 to 1200 s (CP + AC region). The last region is the open circuit region from 1200 s to 1800 s, both CP and AC are stopped. 

Figure 4a shows that the CP potential reaches a relatively stable value in the CP region. With the superimposition of AC after 600 s, the DC potential experiences negative shift. The deviation of DC potential is enlarged by the increase of AC current density. The values of the DC potential under different AC current are recorded in Table 1. When the mandatory polarization of CP and AC is rescinded, the DC potential of the specimens come back to OCP. Figure 4c,e shows the influence of AC on DC potentials when the steady value of CP is −0.95 V_SCE_ and −1.2 V_SCE_, respectively. Different from that of −0.775 V_SCE_, potentials are shifted positively when the AC current is imposed. The higher the AC current density is, the more positive the DC potentials are. When CP and AC are stopped, all DC potentials reach a steady state value of −0.75 V_SCE_, which is the open circuit corrosion potential of X70 steel in the near-neutral pH environment. 

Figure 4b shows the DC current densities under the CP potential of −0.775 V_SCE_. The three regions same as the DC potential measurement are recorded. In the first region from 0 to 300 s, the CP current density is about −15 μA/cm^2^ when there is no AC interference. In the second region, various AC current densities are imposed to the steel while the CP is maintained. When a 1 mA/cm^2^ AC current density is imposed, the cathodic current density decreases to −5.5 μA/cm^2^. When the *i*_AC_ is 3 mA/cm^2^ or higher, anodic current density is detected. The higher the *i*_AC_ applied, the more anodic the current density is. When the mandatory polarization of CP and AC is rescinded, the DC current densities decrease to values that are similar with those in the first region. Figure 4d,f shows the DC current densities recorded on the X70 steel under the CP potential of −0.95 V_SCE_ and −1.2 V_SCE_. It is seen that the DC current densities increase to more cathodic values when AC is applied. Moreover, the higher the AC current density is, the more negative the DC current densities are. When the AC current density increases to 10 mA/cm^2^, the cathodic current densities reach to −109 and −547 μA/cm^2^ under −0.95 V_SCE_ and −1.2 V_SCE_, respectively. When the AC is stopped, the current densities decrease to the values that are similar with those in the first region.

### 3.2. Corrosion Morphology Observation

**Figure 5:**
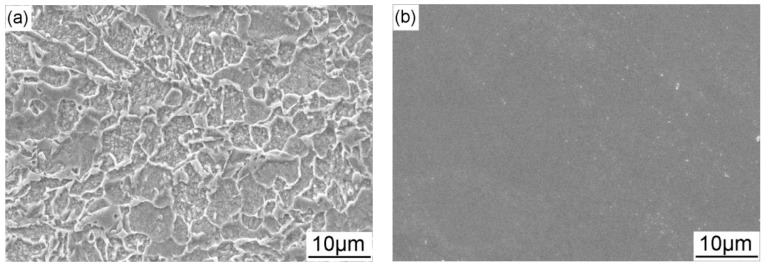
Corrosion morphology of the X70 steel after immersion in the NS4 solution for 24 h without (**a**) and with (**b**) −0.775 V_SCE_ cathodic protection.

The corrosion morphology of X70 steel after immersion in the NS4 solution for 24 h without CP and AC is shown in Figure 5a. It is seen that preferential dissolution occurs in ferrite matrix, and the grain boundaries and carbides remain contact, illustrating the metallographic microstructure of the X70 steel. Figure 5b shows the corrosion morphology of X70 steel under cathodic polarization of −0.775 V_SCE_ for 24 h. It is seen that no corrosion attacks can be detected when the specimen is polarized under this CP potential. Under more negative cathodic protection potentials, the anodic dissolution current densities of the steel become much lower and the corrosion tendency is even lower than that under −0.775 V_SCE_, while the calcium magnesium deposit can be found on the specimen surface.

**Figure 6:**
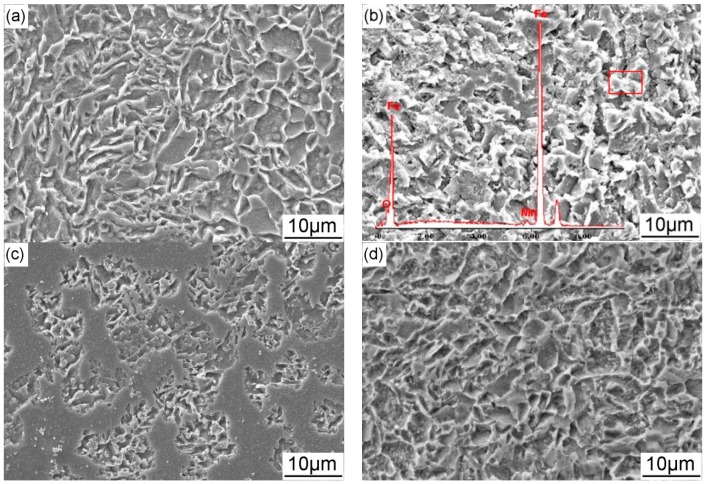

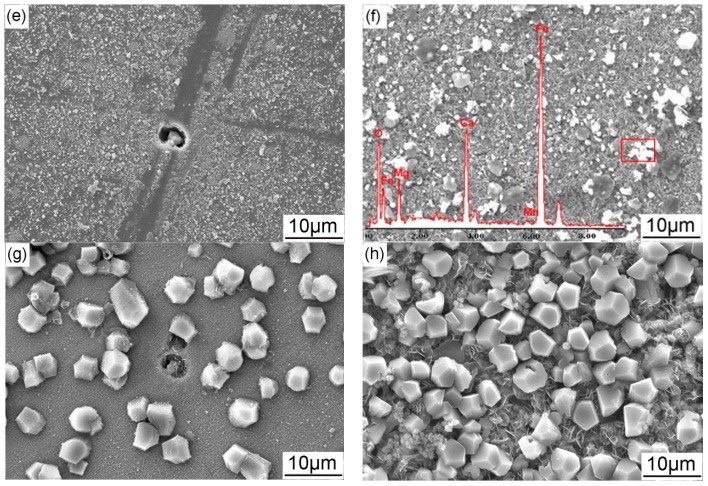
Corrosion morphology of the X70 steel immersed in the NS4 solution for 2 h under the combined effect of CP and AC interference before removing the corrosion products: (**a**) OCP + 3 mA/cm^2^; (**b**) OCP + 10 mA/cm^2^; (**c**) −0.775 VSCE CP + 3 mA/cm^2^; (**d**) −0.775 VSCE CP + 10 mA/cm^2^; (**e**) −0.95 VSCE CP + 3 mA/cm^2^; (**f**) −0.95 VSCE CP + 10 mA/cm^2^; (**g**) −1.2 VSCE CP + 3 mA/cm^2^; and (**h**) −1.2 VSCE CP + 10 mA/cm^2^. The Energy Dispersive Spectrometer (EDS) results of the corrosion products marked in the square shown are illustrated in **b** and **f**.

Figure 6 shows the corrosion morphologies of X70 steel immersed in the NS4 solution for 2 h before removing the corrosion products. Under OCP with the interference of AC, preferential dissolution occurs inside the ferrite grains, while the carbides and the ferrite grain boundaries remain intact as shown in Figure 6a,b. When the AC current is 3 mA/cm^2^, corrosion morphology of the specimen immersed for 2 h is similar with that immersed for 24 h without AC, indicating that AC current accelerates corrosion of X70 steel. With the increase of AC current density, selective dissolution becomes more manifest. Figure 6c,d shows the corrosion morphologies of X70 steel under the combined effect of −0.775 V_SCE_ CP and AC current interference for 2 h in the NS4 solution. When AC current is 3 mA/cm^2^, it is seen that corrosion becomes less severe under the application of −0.775 V_SCE_ CP than that under OCP and only part of the specimen manifests the metallographic microstructure of the steel, which means that −0.775 V_SCE_ CP reduces general corrosion of the steel. When the AC current density increases to 10 mA/cm^2^, corrosion is promoted as compared with that under 3 mA/cm^2^. However, when comparing with that at OCP under the same AC current density, general corrosion is inhibited by −0.775 V_SCE_ CP. Figure 6e,f shows the corrosion morphologies of X70 steel under the combined effect of −0.95 V_SCE_ CP and AC current interference for 2 h in the NS4 solution. The steel surface is covered with deposits which are proved to be calcium and magnesium compounds by EDS analysis. There is no general corrosion under the deposits but some pits are formed accompanying with the deposition of calcium and magnesium compounds. With the increase of AC current density, more calcium and magnesium compounds deposit on the steel surface. Under −1.2 V_SCE_ CP, the AC interference has similar effects as that under −0.95 V_SCE_ as shown in Figure 5g,h, but more crystallographic calcium and magnesium deposits on the steel surface. 

**Figure 7:**
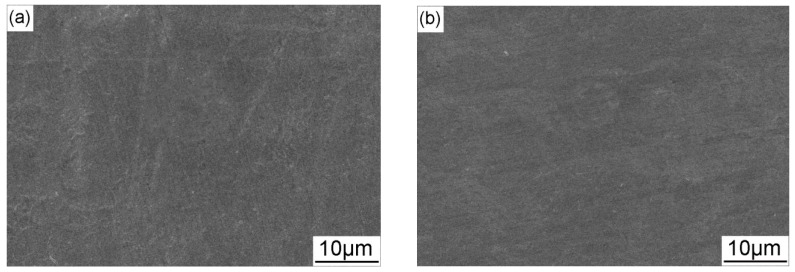

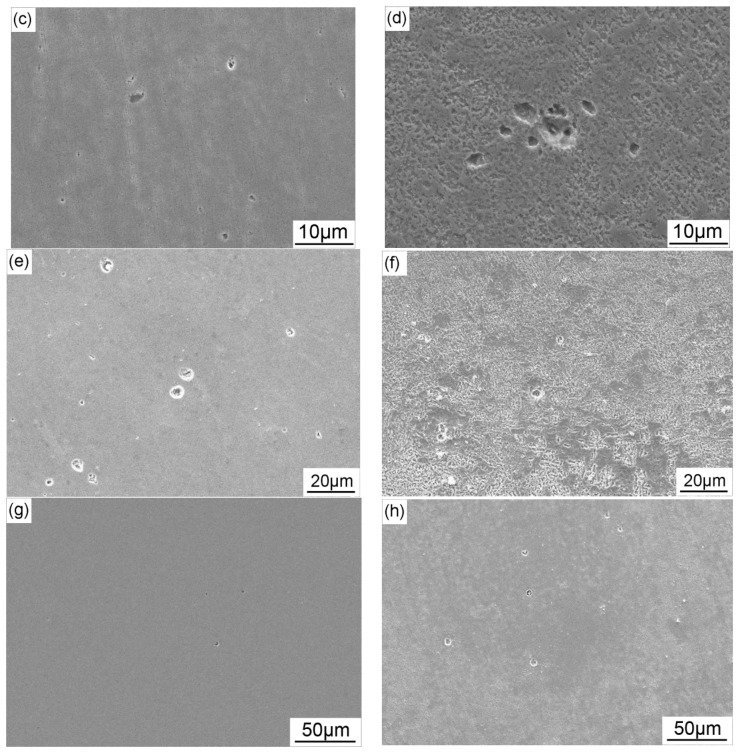
Corrosion morphology of the X70 steel immersed in the NS4 solution for 2 h under the combined effect of CP and AC interference after removing the corrosion products: (**a**) OCP + 3 mA/cm^2^; (**b**) OCP + 10 mA/cm^2^; (**c**) −0.775 VSCE CP + 3 mA/cm^2^; (**d**) −0.775 VSCE CP + 10 mA/cm^2^; (**e**) −0.95 VSCE CP + 3 mA/cm^2^; (**f**) −0.95 VSCE CP + 10 mA/cm^2^; (**g**) −1.2 VSCE CP + 3 mA/cm^2^; and (**h**) −1.2VSCE CP + 10 mA/cm^2^.

Figure 7 shows the corrosion morphologies of X70 steel immersed in the NS4 solution for 2 h after removing the corrosion products. Figure 7a,b shows that no apparent localized corrosion occurs with the interference of 3 and 10 mA/cm^2^ AC current at OCP. Under −0.775 V_SCE_ CP, corrosion pits with the average diameter of 0.75 μm are formed on the specimen when the AC current of 3 mA/cm^2^ is applied (Figure 7c). As the AC current density increases to 10 mA/cm^2^, the diameter of corrosion pits becomes bigger (2.1 μm in average), as shown in Figure 7d. Figure 7e,f shows the corrosion morphologies of X70 steel under the combined effect of −0.95 V_SCE_ CP and AC interference for 2 h after removing the corrosion products. Under this CP potential, bigger corrosion pits (1.6 μm in average) are initiated than that formed under −0.775 V_SCE_ when 3 mA/cm^2^ AC current is applied to the specimen. As the AC current density is increased to 10 mA/cm^2^, severe localized corrosion occurs and some pits with the average diameter of 0.8 μm distribute in isolation. Under the CP potential of −1.2 V_SCE_ and AC current of 3 mA/cm^2^, pit density is much lower than that formed under −0.95 V_SCE_ CP and some metastable pits with low diameters are generated, as shown in Figure 7g. The pit diameter increases to about 0.6 μm when the AC current of 10 mA/cm^2^ is imposed (Figure 7h).

### 3.3. Slow Strain Rate Tension Tests 

**Figure 8:**
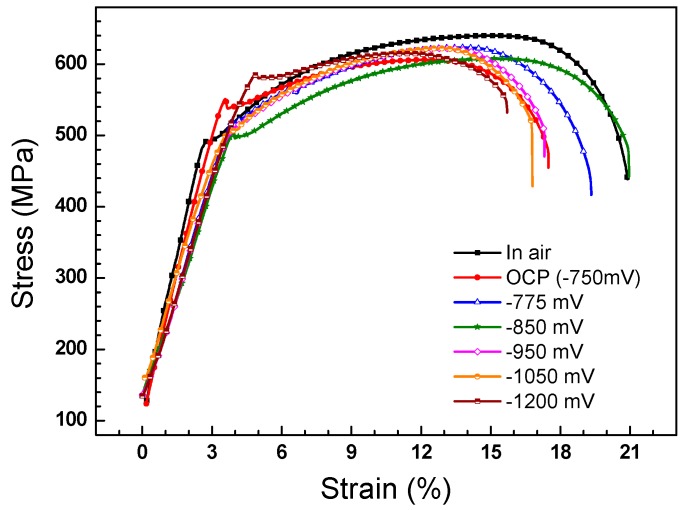
Stress–strain curves of the X70 steel in air and in the NS4 solution under OCP and different CP potentials without AC interference.

**Figure 9:**
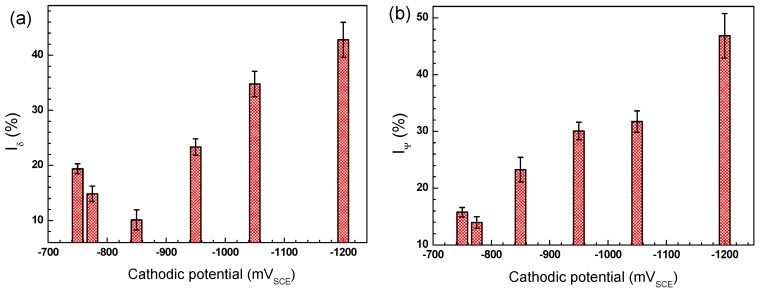
SCC susceptibilities of the X70 steel in air and in NS4 solution under different CP potentials expressed as: the ratio of elongation loss (**a**); and reduction in area (**b**).

Figure 8 shows the stress–strain curves of the X70 steel in air and in the NS4 solution under OCP and different CP potentials without AC interference. In air, the steel exhibits the highest tensile strength and largest elongation. With the application of −0.775 V_SCE_ and −0.85 V_SCE_ CP, the elongation of the steel is higher than that under OCP. However, as the CP potential continues to decrease, the elongation of the steel decreases. Figure 9 shows the SCC susceptibility of the X70 steel under different CP potentials. It is seen that both *R_δ_* (Figure 9a) and *R_ψ_* (Figure 9b) decreases when a −0.775 V_SCE_ CP is applied to the steel, means that the SCC susceptibility of the steel under −0.775 V_SCE_ is lower than that under OCP. The *R_δ_* presents the lowest value under −0.85 V_SCE_, while the *R_ψ_* presents the lowest value under −0.775 V_SCE_. This discordance is due to the hydrogen-induced plasticity (HIP) effect, which has been found on pipeline steels under soft cathodic protection [27]. Under −0.85 V_SCE_, the HIP effect dominates the hydrogen effect on the steel properties as compared with hydrogen embrittlement effect. The small amount of HIP delays the initiation of SCC by releasing the stress intensity and thus increases elongation and decreases *R_δ_* [25]. Meanwhile, the anodic dissolution will still occur and contribute to the SCC process even when the steel is under cathodic protection, resulting in the increase of the *R_ψ_* [28]. That is to say, the SCC sensitivity increases under −0.85 V_SCE_, although the elongation increases. As the CP potential continues to decrease, both *R_δ_* and *R_ψ_* increase, indicating the increase of SCC susceptibility, and the more negative the CP potential is, the more susceptible the steel is to SCC.

**Figure 10:**
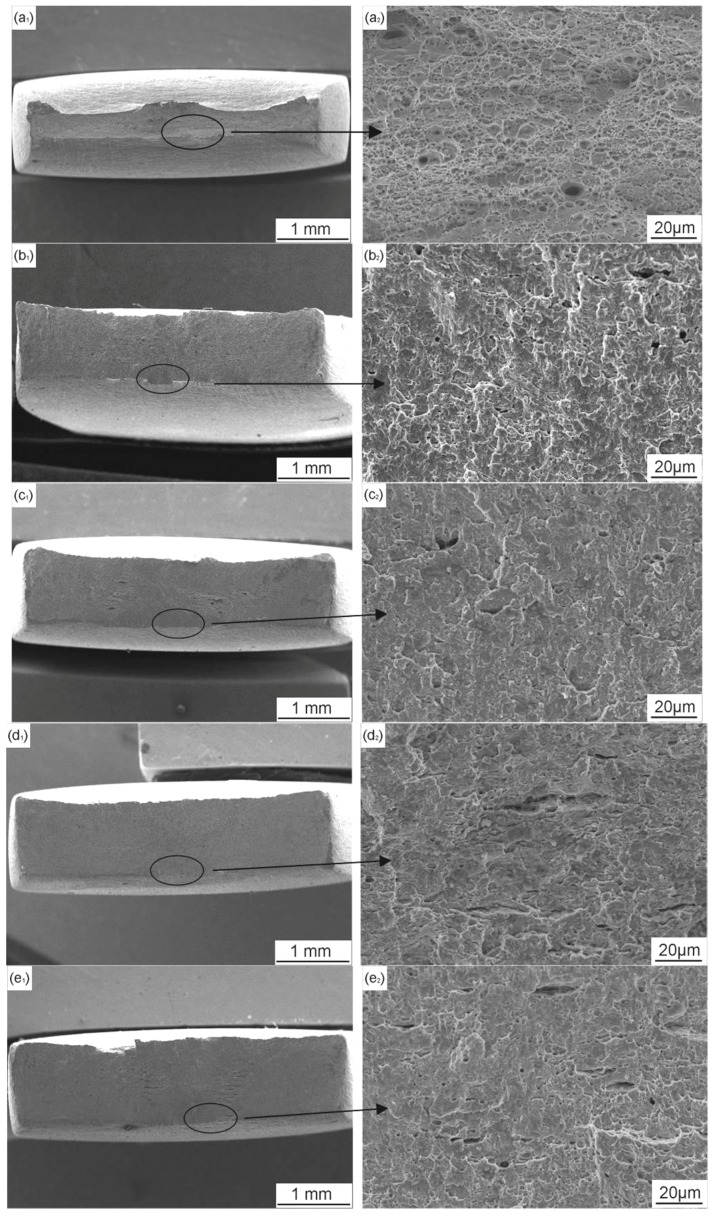

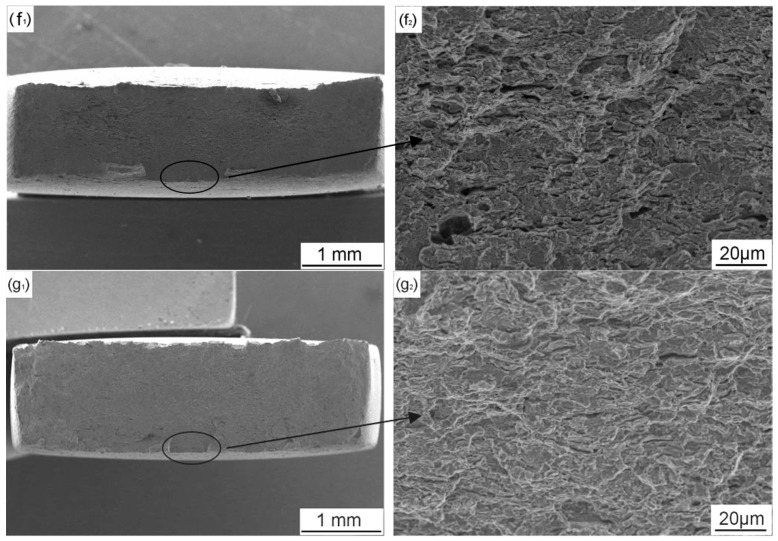
SEM images of the fracture surface of X70 steel: in air (**a**); and in NS4 solution under different CP potentials: (**b**) OCP; (**c**) −0.775 V_SCE_; (**d**) −0.85 V_SCE_; (**e**) −0.95 V_SCE_; (**f**) −1.05 V_SCE_; and (**g**) −1.2 V_SCE_.

Figure 10 shows the morphology of the fracture surface after SSRT tests when the X70 steel is in air, at OCP and at different CP potentials. The surface fractured in air is consisted of a great number of small dimples and microvoids and the necking characteristic is clear (Figure 10a1,a2). When the specimen is tested in near-neutral pH solution under OCP, brittle fracture area is found on the edge of the fracture surface, and the remaining area contains dimples (Figure 10b1,b2). When −0.775 V_SCE_ CP potential is applied, the necking characteristic become more pronounced, but the fracture morphology shows no visible changes (Figure 10c1,c2). Decrease the CP potential to −0.85 V_SCE_, the fracture surface tends to be flat. As the CP potentials continue to decrease, necking of the steel becomes lower and the brittle fracture becomes more manifest, which is consistent with the change of *I_ψ_*. Combining the SCC susceptibility and fracture morphology results, it can be concluded that the −0.775 V_SCE_ provides proper protection for the SCC of X70 steel in near-neutral pH environment. Therefore, the CP potential of −0.775 V_SCE_, which is the industrial-recommended CP criterion (−0.85 V_CSE_), is used to investigate the influence of AC interference on SCC under CP in this work.

**Figure 11:**
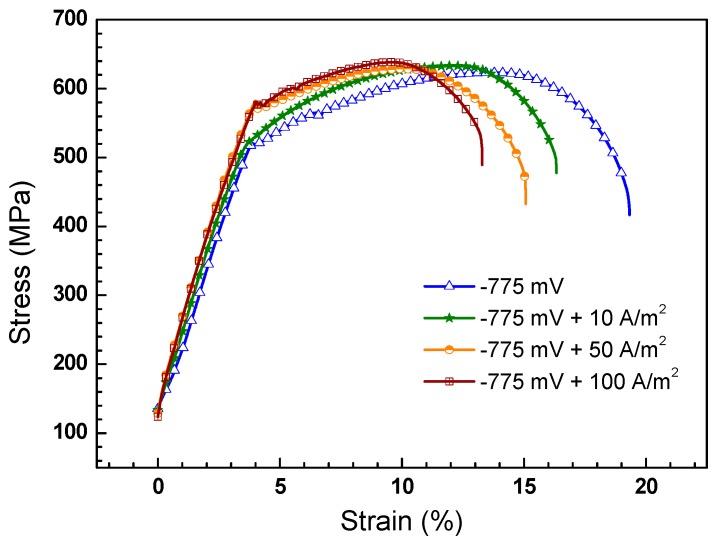
Stress–strain curves of the X70 steel in NS4 solution under −0.775 V_SCE_ CP with application of different AC current densities.

**Figure 12:**
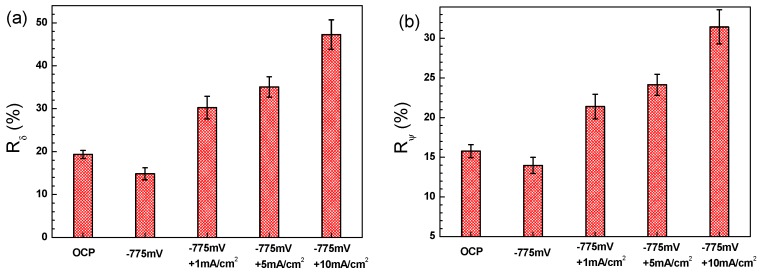
SCC susceptibilities of the X70 steel in NS4 solution under −0.775 V_SCE_ CP with application of different AC current densities expressed as: the ratio of elongation loss (**a**); and reduction in area (**b**).

Figure 11 shows the stress–strain curves of X70 steel measured in the NS4 solution under the combined effect of −0.775 V_SCE_ CP and AC interference. The highest elongation is observed when the specimen is tested without AC interference, and it decreases significantly with increase of AC current density, suggesting that AC decreases SCC resistance of X70 steel. Figure 12 shows the SCC susceptibility of the X70 steel under −0.775 V_SCE_ CP and superimposed AC interference. It is seen that the *R_δ_* (Figure 12a) and *R_ψ_* (Figure 12b) present the same changing tendency as the experimental condition varies. Both increase as the AC current is applied. Combined with Figure 9, the experimental results illustrate that AC enhances the SCC susceptibility of X70 steel, and the higher the AC current is, the more susceptible the steel is to SCC. It is worth noting that even a small AC current of 1 mA/cm^2^ could promote the SCC process.

**Figure 13:**
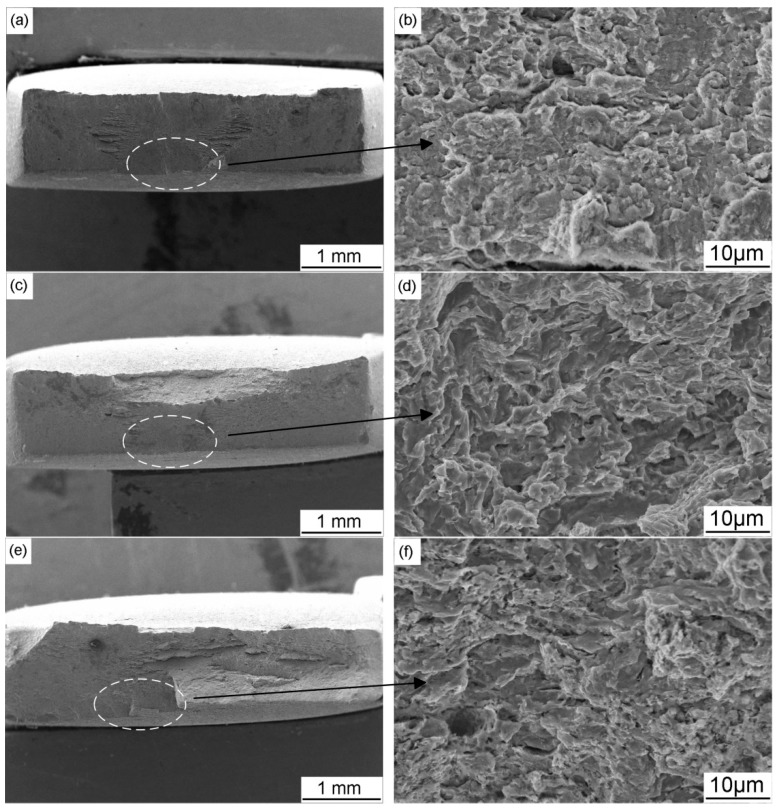
SEM images of the fracture surface of X70 steel in NS4 solution under −0.775 V_SCE_ CP with application of different AC current densities: (**a**,**b**) 1 mA/cm^2^; (**c**,**d**) 5 mA/cm^2^; and (**e**,**f**) 10 mA/cm^2^.

Figure 13 shows the SEM views of the fracture surface morphology after SSRT tests when the X70 steel is under combined CP and AC interference. When AC current is superimposed, large brittle fracture zones can be observed. The micro-morphology of the fracture surface shows apparent brittle characteristics when the AC current is 5 mA/cm^2^ (Figure 13c,d) and 10 mA/cm^2^ (Figure 13e,f), which also indicates that AC decreases the resistance of X70 steel to SCC when it is under cathodic protection.

## 4. Discussion

It is seen that both the corrosion dissolution (Figure 5) and stress corrosion cracking process (Figure 8) are inhibited by −0.775 V_SCE_ CP when there is no AC interference, while localized corrosion such as pitting and SCC are promoted with the superimposition of AC. The following sections will discuss the mechanism of the AC-induced variation of the corrosion process.

### 4.1. Influence of AC on CP Potential and Current

It has been acknowledged [29] that DC potential is closely related to the quantity of electrons on the electrode surface. Thus, the shifting direction of the DC potential is decided by the relative magnitude of anodic and cathodic current densities of the electrode. There are three circumstances according to the electrons produced by anodic reactions and consumed by cathodic reactions, which has also been reported by Xu and Cheng [20]:

(I) $\left|i_{a}+i_{e}\right|>\left|i_{c}\right|$, that is the sum of the free electrons from the externally applied current and the anodic reaction is more than that consumed by the cathodic reaction. In this case, anodic dissolution is promoted and redundant electrons remain in the steel surface, leading to the negative shift of DC potential.
(II) $\left|i_{a}+i_{e}\right|=\left|i_{c}\right|$, that is the sum of the supply rate of electrons by external current and anodic reaction is balanced by the consumption rate. Accordingly, no change of DC potential will be observed.
(III) $\left|i_{a}+i_{e}\right|<\left|i_{c}\right|$, that is the supplying rate of the free electrons is smaller than the cathodic consumption rate. As a result, the cathodic reaction is promoted and DC potential is shifted to the positive direction.

Theoretically, the DC potential gradually stabilized at open circuit potential when there are no external current sources. With the application of CP, new steady state of DC potential lower than the open circuit potential, which is called cathodic polarization, is reached because of the constant input of excess electrons. With the superimposition of AC, the steel is anodically and cathodcially polarized periodically. Under a weak cathodic protection potential of −0.775 V_SCE_, the cathodic polarization is enhanced by AC and more electrons are provided by external current sources, which causes the negative shift of DC potential in the CP + AC region (Figure 4a). When the CP potential is maintained at −0.95 V_SCE_ or −1.2 V_SCE_, far more electrons are provided than that of −0.775 V_SCE_, resulting in the large deviation of the electrode from natural equilibrium state of OCP. In this case, the electrode has strong tendency to come back to the equilibrium state and only a slight disturbance of AC can cause the positive shift of DC potential (Figure 4c,e).

It has been reported that the process occurring during anodic half-cycle of the AC signal is not completely reversed during the cathodic half-cycle [7], meaning that a larger anodic current density than the cathodic current density is induced by AC when there is no CP. When the CP potential is −0.775 V_SCE_, the AC interference has similar effect with that of OCP. Anodic dissolution reaction rate is promoted, resulting in the increase of DC current from −15 to −6 μA/cm^2^ when 1 mA/cm^2^ *i*_AC_ is superimposed to the electrode (Figure 4b). When the *i*_AC_ is 3 mA/cm^2^ or higher, the anodic reaction is accelerated more heavily. As a result, the current densities become anodic in CP + AC region. With the application of −0.95 V_SCE_ or −1.2 V_SCE_ CP, the electrode has the tendency to come back to the equilibrium state of OCP when there is another external disturbance. Thus, more electrons are needed to maintain the cathodic polarization potential of the electrode with the disturbance of AC. More electrons mean higher cathodic current density. Therefore, DC current density is cathodic and the cathodic current density increases with the application of AC, as shown in Figure 4d,f. 

### 4.2. Influence of AC on Corrosion Morphology

When there is no AC interference, no corrosion is detected under the application of CP (Figure 5). While when AC current is superimposed, the steel is corroded, even though it is cathodically protected (Figure 6). In the initial period of immersion under AC interference, the surface of the steel shows a metallurgic microstructure-like morphology. It reveals that α ferrite grains are prone to dissolve, while the carbides and the grain boundaries are away from attacking, attributing to the micro-galvanic effect between different phases in the steel [30]. Atrens et al. [31] found that grain boundaries of X70 steel had higher carbon concentration than ferrite grains. Liu et al. [32] characterized the local electrochemical activity of grains and grain boundaries of X70 steel and found that the grains were anodic relative to the grain boundaries. Tsuyoshi [33] measured the corrosion potential of iron and various carbides in different electrolytes and reported that all the tested carbides including cementite presented nobler corrosion potential than iron. In conclusion, carbon-rich structures of X70 steel act as cathode in the process of corrosion in the near-neutral pH solution, resulting in the corrosion morphology similar with the microstructure. 

Under a CP potential of −0.775 V_SCE_, corrosion is promoted remarkably by the AC interference (Figure 6c,d). As aforementioned in Section 4.1, the anodic dissolution is enhanced when the AC current is applied. Even though the measured DC current is a cathodic current of −6 μA/cm^2^ when the *i*_AC_ is 1 mA/cm^2^, anodic dissolution also occurs (Figure 8). Moreover, dissolution of the steel is aggravated by the increase of AC current density. This is in accordance with previous studies [34,35], which also found the occurrence of AC corrosion even if the pipeline steels are cathodically protected. More negative CP potentials are needed to decrease the corrosion rate of the steel under AC interference [20].

Under the cathodic protection of −0.95 V_SCE_ and −1.2 V_SCE_, cathodic reaction is promoted when the AC current is imposed as previously analyzed. However, pitting corrosion still occurs even though the cathodic current is kept at a quite high value (Figure 6e,g). The AC current densities used in this work are lower than the threshold to initiate pitting, which is found to be 20 mA/cm^2^ by previous study [15], in the near-neutral pH solution. Therefore, no pit forms on the steel surface at OCP (Figure 7a,b) and it can be speculated that the combined effect of CP and AC current is responsible for the initiation of corrosion pits. Under CP condition, the steel is protected from corrosion. When the AC current is imposed, the specimen is anodically and cathodcially polarized periodically [36]. If the AC current is efficiently high, e.g., 3 mA/cm^2^, the short-term anodic polarization can be achieved. Therefore, anodic current is generated, resulting in the local anodic dissolution of the active sites, such as the scratches, inclusions, dislocation slip steps and dislocation emergence points [17,37]. Because the frequency of AC current is 50 Hz, the anodic polarization, which is followed by the relatively long-term cathodic polarization, could be only kept for a short time. Corrosion pits are initiated at the active sites and other areas are not attacked (Figure 7e,f). When the CP potential is efficiently negative, e.g., −1.2 V_SCE_, the application of 3 mA/cm^2^ AC current can only generate a small anodic current. In this case, the metastable pits are formed (Figure 7g). As the AC current density increases to 10 mA/cm^2^, higher anodic current is induced and corrosion pits with bigger diameters are initiated (Figure 7h). 

### 4.3. Influence of AC on Stress Corrosion Cracking

In the near-neutral pH solution, both dissolution of the steel and hydrogen ingress into the steel are acknowledged to be involved in the SCC process of pipeline steels [38,39,40]. That is to say, SCC of pipeline steels is mix-controlled by anodic dissolution (AD) and hydrogen embrittlement (HE). Therefore, AC-induced increase of SCC susceptibility under CP can be explained by the variation of anodic dissolution and hydrogen evolution processes. 

As discussed in Section 4.2, ferrite grains are preferentially attacked when the X70 steel is immersed in the testing solution. Therefore, the electrochemical driving force for cracks to propagate within a grain is higher than that at grain boundaries [32,41], resulting in the transgranular stress corrosion cracking (TGSCC). When the X70 steel is protected by a CP potential of −0.775 V_SCE_, the anodic reaction is inhibited. There are no preferential sites for SCC cracks to initiate and the SCC susceptibility decreases in comparison with OCP. Upon the application of AC current, the anodic dissolution is enhanced. That is, the ferrite grains are more severely corroded. Thus, stress corrosion cracks more easily propagate once initiated within the grains and the SCC is promoted.

On the other hand, the AC-induced hydrogen evolution also involves in the SCC process. Under the CP potential of −0.775 V_SCE_, the cathodic hydrogen evolution on X70 steel is weak. The small amount of hydrogen induces the hydrogen-facilitated plasticity and increases the elongation of X70 steel (Figure 9). When the AC current is superimposed, even though the measured DC current is cathodic, such as *i*_AC_ = 1 mA/cm^2^, the instantaneous electrode potential induced by both CP and AC polarization can reach a large negative value [42]. If the instantaneous large negative electrode potential is lower than the threshold of hydrogen evolution, H atoms can be produced due to the reduction of H_2_O and HCO_3_^−^ [9,43]:

(3) $$H_{2}O+e\to {H(\mathrm{ads})+\mathrm{OH}}^{-}$$

(4) $${\mathrm{HCO}}_{3}{}^{-}+e\to {H(\mathrm{ads})+\mathrm{CO}}_{3}^{2^{-}}$$

The H atoms has great impact on SCC by absorbing on the electrode surface and ingressing into the steel [44], which can be described in two aspects. Firstly, hydrogen can induce alteration of electrochemical kinetic parameters, such as chemical potential and exchange current density of corrosion reactions, which subsequently enhances the anodic dissolution of the steel [45]. With the increase of AC current density, more hydrogen atoms are produced, resulting in the increase of anodic dissolution current density [15]. In addition, the existence of hydrogen can promote local plastic deformation, which is believed to be the key factor for the initiation and propagation of SCC in near-neutral pH environment [28]. The enhancement of local plastic deformation leads to brittle fracture of the steel (Figure 13). Consequently, SCC susceptibility is increased significantly by the application of AC (Figure 12) due to the production of hydrogen. When the *i*_AC_ is increased, the cathodic current would be higher due to the more negative potential induced by AC [15], and thus the SCC susceptibility increases.

### 4.4. Implication on the CP Effectiveness of Pipeline in the Field

According to previous studies, AC current density is believed to be the primary factor that affect AC corrosion of pipeline steels [3]. For the pipelines without CP, an AC current density criterion for AC corrosion was educed. When *i*_AC_ is below 3 mA/cm^2^, there is probably no risk of AC corrosion. When *i*_AC_ is between 3 and 10 mA/cm^2^, corrosion is possible, since conventional criteria are not reliable. At AC current densities in excess of 10 mA/cm^2^, corrosion damage is to be expected. The European standard EN 15280 [46] also proposed that AC corrosion mitigation was achieved by reducing *i*_AC_ below 3 mA/cm^2^ and maintaining CP current density below 0.1 mA/cm^2^ if *i*_AC_ was higher than 3 mA/cm^2^. Büchler [23,24] conducted extensive laboratory and field tests and reported that for current density values determined on coupons one of the criteria below must be met: (i) average *i*_AC_ < 3 mA/cm^2^; (ii) average *i*_DC_ < 0.1 mA/cm^2^; or (iii) *i*_AC_/*i*_DC_ < 3. However, most of the previous works focus on the corrosion rate without considering the SCC susceptibility. In this work, under the CP potential of −0.775 V_SCE_, the AC current as low as 1 mA/cm^2^ can increase the SCC susceptibility to a higher value than OCP condition (Figure 12). It is speculated that SCC susceptibility can be increased by AC under a more negative CP potential. As has been analyzed, the imposed AC can facilitate both the cathodic and the anodic reactions, especially the former. On the one hand, the accelerated hydrogen evolution reactions will generate more hydrogen atoms, contributing to the AD and HE mix-controlled near-neutral pH SCC [15]. On the other hand, the accelerated anodic dissolution will lead to generation of corrosion pits, which usually become the initiation sites for stress corrosion cracks [47], and thus the SCC susceptibility will increase. Therefore, in field operation, when the CP criteria under AC interference is used, the SCC risk should be considered.

## 5. Conclusions

In the present study, AC interference on corrosion and SCC of X70 steel under cathodic protection was investigated. The main conclusions can be drawn as follows:

(1) Both corrosion and SCC are inhibited by −0.775 V_SCE_ CP without AC interference. Further decrease the CP potential to more negative values, resistance of the steel to SCC decreases.
(2) Under the CP of −0.775 V_SCE_, DC potential shift to the negative direction with the superimposition of AC. Meanwhile, the cathodic DC current decreases and shift to the anodic direction. When the AC current is 3 mA/cm^2^ or higher, the DC current becomes anodic. The higher the *i*_AC_ applied is, the more anodic the current density is.
(3) Under the CP of −0.95 V_SCE_ and −1.2 V_SCE_, the applied AC current promotes the cathodic reaction and results in a positive shift of the DC potential and an increase of the cathodic current. Local anodic dissolution occurs on the cathodically protected steel under AC interference, attributing to the generated anodic current in the positive half-cycle of the AC current. The active sites on the steel surface are preferentially attacked and corrosion pits are initiated.
(4) AC enhances the SCC susceptibility of X70 steel under CP, attributing to the promotion of anodic dissolution and hydrogen evolution. Even a small AC current can degrade the SCC resistance. Thus, the SCC susceptibility of pipeline steel should be considered when the CP standard under AC interference is established.

## Figures and Tables

**Table 1:** Steady-state DC potential and DC current density of X70 pipeline steel under the combined effect of CP and AC interference in NS4 solution.

AC Current Density	−0.775 V_SCE_		−0.95 V_SCE_		−1.2 V_SCE_	
	DC Potential (V_SCE_)	DC Current (mA/cm^2^)	DC Potential (V_SCE_)	DC Current (mA/cm^2^)	DC Potential (V_SCE_)	DC Current (mA/cm^2^)
0 mA/cm^2^	−0.775	−0.015	−0.950	−0.044	−1.200	−0.271
1 mA/cm^2^	−0.781	−0.006	−0.905	−0.063	−1.185	−0.292
3 mA/cm^2^	−0.820	0.022	−0.887	−0.081	−1.163	−0.365
5 mA/cm^2^	−0.846	0.050	−0.878	−0.087	−1.144	−0.391
10 mA/cm^2^	−0.871	0.081	−0.868	−0.109	−1.095	−0.547
